# 中国首次复发多发性骨髓瘤诊治指南（2026年版）

**DOI:** 10.3760/cma.j.cn121090-20251130-00559

**Published:** 2026-03

**Authors:** 中华医学会血液学分会浆细胞疾病学组 中国医师协会多发性骨髓瘤专业委员会

## Abstract

多发性骨髓瘤仍是一种不能治愈的恶性肿瘤，首次复发后的治疗选择对于患者的预后非常重要。近三年我国获批多种治疗多发性骨髓瘤的新药，本次指南在第一版指南的基础上进行了更新，包括引入早期复发尤其是功能性高危的概念，对首次复发后的治疗时机、治疗方案尤其是免疫治疗的选择进行了更新。

## Abstract (en)

Multiple myeloma（MM）remains an incurable malignancy, and treatment choices after the first relapse are crucial for patients' prognosis. Over the past three years, multiple novel drugs for MM have been approved in China. Based on the first edition, this guideline has been updated to introduce the concept of early relapse, especially functional high-risk, and revise the timing of treatment, therapeutic regimens, particularly the selection of immunotherapy, for patients after the first relapse.

一、概述

多发性骨髓瘤（MM）目前仍是一种不能治愈的疾病，大部分患者经最初治疗达到某种程度缓解后仍不可避免复发[1]–[2]。首次复发患者机体免疫功能尚可，大多未产生多药耐药，脏器功能可耐受多种治疗方法，经恰当治疗可再次达到较深程度缓解，从而获得较长的无进展生存（PFS）期和总生存（OS）期。因此，首次复发后的治疗选择尤为重要。本指南在第一版《中国首次复发多发性骨髓瘤诊治指南（2022年版）》[3]的基础上，结合近年来该领域的发展、文献证据与专家共识，对推荐内容进行修订，并增加相关内容。修订要点如下：①根据首次复发的时间提出早期复发和功能性高危（FHR）的定义。②阐述近三年国家药品监督管理局（NMPA）新药获批情况，包括双特异性抗体（BsAb）如特立妥双抗、埃纳妥双抗、塔奎妥双抗；嵌合抗原受体T细胞（CAR-T细胞）如伊基奥仑赛、泽沃基奥仑赛；第二款CD38单克隆抗体艾沙妥昔单抗；我国创新一类新药埃普奈明等及相关重要临床试验数据。③更新首次复发MM患者的治疗推荐。

二、首次复发的定义

复发一般是指完全缓解（CR）后复发及微小残留病（MRD）阴性后复发，进展指获得部分缓解（PR）及非常好的部分缓解（VGPR）疗效后出现指标增长达到一定标准，临床中往往把复发和进展列在一起。MM患者接受规范的一线治疗达到PR及以上疗效后出现疾病进展/复发定义为首次复发。复发的类型可分为临床复发、生化复发、CR后复发、MRD阴性转为阳性复发；根据复发时的生物学特征及疾病进展速度可分为侵袭性复发和非侵袭性复发；根据M蛋白增长的速度，生化复发又分为快速生化复发和缓慢生化复发[1],[4]–[7]。

（一）复发类型

1. 临床复发：符合以下一项或多项：①出现新的骨病变（骨质疏松性骨折除外）或软组织浆细胞瘤；②明确的已有的浆细胞瘤或骨病变增加（可测量病变最大垂直径乘积之和增加≥50％且绝对值≥1 cm）；③高钙血症（校正血清钙>2.75 mmol/L）；④HGB下降≥20 g/L（排除治疗或其他可降低HGB的因素）；⑤自MM治疗开始，血肌酐上升≥176.8 µmol/L（2 mg/dl），排除其他可能导致肌酐上升的因素；⑥血清M蛋白相关的高黏滞综合征。

2. 生化复发：符合以下条件中任意一项，且暂无MM相关器官功能损害或症状。①血清M蛋白升高≥25％（升高绝对值需≥5 g/L）；②尿M蛋白升高≥25％（升高绝对值需≥200 mg/24 h）；③若血清和尿M蛋白无法检出，受累与非受累血清游离轻链（FLC）差值增加≥25％（增加绝对值需>100 mg/L）；④骨髓浆细胞比例增加绝对值≥10％。

3. CR后复发：按照国际骨髓瘤工作组（IMWG）疗效评判标准证实已达CR的患者，血或尿M蛋白再次出现或骨髓浆细胞比例≥5％。移植或不移植患者经系统治疗后曾达CR，短时间内再次出现M蛋白时应注意和免疫重建鉴别。早期免疫重建M蛋白多为IgG型，不会导致其他免疫球蛋白进一步下降，复查骨髓无克隆性浆细胞，数月后可自行消失，此现象提示预后较佳[8]。

4. MRD阴性后复发：连续两次监测失去MRD阴性状态（证实存在克隆性浆细胞或影像学提示MM复发）。MRD敏感性规定至少在10^−5^水平，建议应用新一代流式细胞术（NGF）或二代测序（NGS）进行检测。影像学检查建议应用全身PET-CT评估。

5. 特殊形式复发：部分患者以髓外浆细胞瘤的形式复发，髓外浆细胞瘤的定义见上述临床复发中的“①”或“②”。中枢神经系统受累的复发是一种特殊形式的髓外复发。还有部分MM患者以浆细胞白血病的形式复发，外周血涂片浆细胞比例≥5％称为继发性浆细胞白血病[9]。

（二）早期复发的定义

对于早期复发的时间界定目前并未统一，一般认为，在目前标准三药诱导治疗开始后的18个月内和（或）一线自体造血干细胞移植（auto-HSCT）后的12个月内复发称为早期复发。部分早期复发患者在诊断时无任何提示高危的临床特征和不良细胞遗传学等标志，称为FHR[10]–[13]。

（三）侵袭性复发和非侵袭性复发

根据复发的生物学特征和疾病进展速度可分为侵袭性复发和非侵袭性复发，具备下列任意一项者定义为侵袭性复发：①新出现的不良细胞遗传学异常，如17p−、1q21+和亚二倍体；②血肌酐正常情况下高β_2_微球蛋白（≥5.5 mg/L）或低白蛋白（<35 g/L）；③出现髓外浆细胞瘤；④LDH（与MM相关）大于正常值上限；⑤缓解期短或治疗中出现疾病进展；⑥出现侵袭性临床表现，包括快速出现症状，实验室、影像学或病理检查发现广泛的疾病进展、疾病相关的器官功能不全；⑦外周血涂片发现浆细胞（流式细胞术证实为克隆性浆细胞）；⑧免疫球蛋白类型转化（轻链逃逸，浆细胞分泌活性降低）。以上条件都不具备的患者考虑为非侵袭性复发[1]。

三、首次复发需要完善的检查

如考虑复发，建议完善表1中所列项目。其中，细胞遗传学和全身PET-CT非常重要，即使新诊断时存在高危细胞遗传学异常，复发后仍需再次评估，以确定是否获得更高比例的高危细胞遗传学异常或增加预后更差的细胞遗传学异常（如双打击、三打击等），全身PET-CT主要用于评估髓外病变。即使初诊时无髓外表现，复发时往往有出现髓外病变的可能。建议有条件者增加MM相关基因突变检查。

**表1:** 多发性骨髓瘤首次复发需要完善的检查

项目	内容
血液检查	血常规、肝肾功能（包括肌酐、白蛋白、球蛋白、乳酸脱氢酶、碱性磷酸酶、尿酸）、电解质（包括钙离子）、凝血功能、β_2_微球蛋白、外周血涂片（浆细胞比例，必要时行外周血流式细胞术检查）；血M蛋白相关检查：血清免疫球蛋白定量、血清蛋白电泳、血免疫固定电泳、血清游离轻链
尿液检查	尿常规、24 h尿蛋白定量、24 h尿轻链检测、尿蛋白电泳、尿免疫固定电泳
骨髓检查	骨髓细胞涂片±活检（浆细胞比例）；流式细胞术检查：CD38/CD138/CD56/CD19/CD20/cκ/cλ；荧光原位杂交（FISH）：包括但不限于以下内容：17p缺失、13q14缺失、1q21获得/扩增、1p32缺失、t（4;14）、t（6;14）、t（11;14）、t（14;16）、t（14;20）；有条件者行相关基因突变的测定
影像学检查	全身PET-CT；如无法行全身PET-CT，建议行MRI或全身低剂量CT或全身X线平片（包括扁骨和长骨）
其他（器官功能评估）	心电图、氨基末端脑钠肽前体（NT-proBNP）、肌钙蛋白T（TnT）/肌钙蛋白I（TNI）、超声心动图、胸部CT、腹部B超等，必要时肺功能检测

四、首次复发治疗选择需考虑的因素

1. 患者一般状况的评估：复发时的年龄、合并疾病（如糖尿病、高血压、冠心病、慢性阻塞性肺疾病等）、体能状态评分（ECOG评分）或虚弱评分［推荐IMWG老年评估（GA）评分］[14]、经济状况、个人意愿。重要脏器功能评估如心脏功能、肺功能、肝功能、肾功能等。

2. 复发后疾病状态的评估：需要评估MM复发的类型和特征（临床复发、生化复发、CR后复发、MRD阴性转为阳性复发、是否为侵袭性复发等），全身PET-CT是否合并髓外病变，细胞遗传学评估复发后的获得性改变等。

3. 既往一线治疗情况的评估：

（1）一线治疗的方式和药物：一线治疗是否行auto-HSCT，一线治疗应用药物情况，如蛋白酶体抑制剂（PI）、免疫调节剂（IMiD）、CD38单克隆抗体等，充分评估患者在一线治疗期间药物暴露和可能存在的耐药或难治情况。

（2）一线治疗的缓解深度及持续时间：需评估一线治疗获得的最佳疗效及疗效持续时间，复发与第一次行auto-HSCT的间隔时间，距离首次有效诱导方案治疗的时间，评估是否早期复发。

（3）一线治疗相关毒性及可能影响：①如一线治疗出现硼替佐米相关的1级伴疼痛或2级以上周围神经炎，硼替佐米不再推荐继续应用；②卡非佐米有心脏、肾脏毒性；③CD38单克隆抗体增加感染风险；④对于发生过深静脉血栓的患者，尽可能避免再次应用含IMiD的方案，如确需继续使用，建议予标准的预防血栓治疗[15]；⑤6个月内使用过大剂量烷化剂及苯达莫司汀可能导致T细胞功能受损，可能会影响T细胞采集及CAR-T细胞扩增；⑥患者接受过放射治疗可能会影响造血干细胞采集；⑦6个月内使用过BCMA靶向治疗可能会影响BCMA CAR-T细胞的疗效[16]。

五、首次复发的治疗选择

（一）首次复发的治疗目标和治疗时机选择

1. 治疗目标：首次复发的治疗目标是获得最大程度的疾病缓解，延长PFS期。

2. 治疗时机的选择：不是所有MM患者在复发后需马上启动治疗，应根据复发形式和复发特征作出选择。如为临床复发则需即刻启动治疗。如为快速生化复发或符合侵袭性复发的标准，建议尽早启动治疗。对于M蛋白增长缓慢的生化复发患者，建议可先观察，每2～3个月评估1次。但目前倾向于即使是较慢的生化复发，早期启动复发治疗能使患者获益[17]–[18]。对于早期复发（包括FHR）患者，鉴于该类型患者预后差，对常规治疗效果欠佳，一旦复发，无论何种形式，均建议早期积极干预。MRD阴性转为阳性复发是否需要干预尚不明确，目前的处理以观察为主，也可开展临床研究。

（二）我国首次复发MM可选择的药物和治疗方法

近几年，一系列新药相继在我国获批用于治疗MM，主要包括：①免疫治疗：CD38单克隆抗体、CAR-T细胞治疗、BsAb；②新一代IMiD、新一代PI；③其他作用机制的药物：塞利尼索、埃普奈明。具体药物及获批情况见表2。

**表2:** 目前中国已获批治疗多发性骨髓瘤的新药及指南推荐[19]–[20]

药物	FDA	ESMO指南	NCCN指南	NMPA	中国获批时间
达雷妥尤单抗	NDMM、RRMM	NDMM，首次复发、多线复发	NDMM，前线复发、多线复发	NDMM，首次复发	2019年10月
卡非佐米	RRMM	首次复发、多线复发	NDMM，前线复发	RRMM（既往接受至少两种治疗，包括PI和IMiD）	2021年7月
泊马度胺	RRMM	首次复发、多线复发	前线复发	RRMM（既往接受至少两种治疗，包括一种PI和来那度胺）	2020年11月
塞利尼索	RRMM	首次复发、多线复发	前线复发、多线复发	RRMM（既往接受至少一种PI、一种IMiD及CD38单克隆抗体）	2021年12月
艾沙妥昔单抗	NDMM、RRMM	首次复发、后续复发	NDMM，前线复发	NDMM，首次复发	2025年1月
特立妥双抗	RRMM	临床试验	多线复发	RRMM（既往接受至少三线治疗，包括一种PI、一种IMiD和CD38单克隆抗体）	2024年11月
埃纳妥双抗	RRMM	临床试验	多线复发	RRMM（既往接受至少三线治疗，包括一种PI、一种IMiD和CD38单克隆抗体）	2025年3月
塔奎妥双抗	RRMM	临床试验	多线复发	RRMM（既往接受至少三线治疗，包括一种PI、一种IMiD和CD38单克隆抗体）	2025年2月
伊基奥仑赛^a^	/	/	/	RRMM（既往接受至少三线治疗，包括至少一种PI和一种IMiD）	2023年7月
泽沃基奥仑赛^a^	/	/	/	RRMM（既往接受至少三线治疗，包括至少一种PI和一种IMiD）	2024年3月
埃普奈明^a^	/	/	/	RRMM	2023年11月

**注** FDA：美国食品药品监督管理局；ESMO：欧洲肿瘤内科学会；NCCN：美国国立综合癌症网络；NMPA：国家药品监督管理局；NDMM：初诊多发性骨髓瘤；RRMM：复发难治多发性骨髓瘤；PI：蛋白酶体抑制剂；IMiD：免疫调节剂；/：无内容；^a^未在国外获批

（三）我国首次复发MM患者的治疗推荐

1. 治疗原则：早期复发患者在所有初诊患者中占10％～12％，无论初诊时是否具备高危细胞遗传学或高危临床特征，均应被视为高危。早期复发患者对于常规治疗效果欠佳，推荐进入临床试验。如无合适临床试验，首选CAR-T细胞治疗或BsAb治疗。如无条件进行CAR-T细胞治疗和BsAb治疗，根据既往用药和耐药情况选择合适的方案。

对于非侵袭性复发患者，根据一线是否已行auto-HSCT及是否在初次诱导治疗时冻存造血干细胞决定是否采用晚期移植或挽救性移植。诱导治疗方案根据既往用药情况可选择既往有效的方案或新一代PI、新一代IMiD、单克隆抗体或不同作用机制的药物联合，多线复发后再考虑其他治疗，包括CAR-T细胞或BsAb等。侵袭性复发患者的治疗原则建议与早期复发相同，即选择CAR-T细胞治疗和（或）BsAb治疗，或进入临床试验。如无条件进行上述治疗，可根据患者既往用药及可能耐药情况选择治疗方案。

2. 特殊情况复发

（1）髓外复发：如患者以髓外复发（包括浆细胞白血病）为主，建议进入临床试验，如无合适临床试验，首选CAR-T细胞治疗，两种BsAb联合治疗对于髓外病灶可能有一定的疗效[21]。如患者无条件选择CAR-T细胞和BsAb治疗，可选择含苯达莫司汀的联合方案、含塞利尼索的联合方案、含细胞毒性药物的多药联合方案治疗结合局部放疗等。我国多中心临床研究结果显示，应用埃普奈明联合KT-DECP方案（卡非佐米+沙利度胺+地塞米松+依托泊苷+环磷酰胺+顺铂）对髓外复发患者有一定疗效[22]。孤立髓外复发患者完成局部治疗后应密切随访。髓外复发特别是血行播散型预后较差，中枢神经系统髓外浸润患者预后更差，需要使用可穿透血脑屏障的药物包括泊马度胺、塞利尼索等。

（2）虚弱患者的治疗选择：建议按照IMWG GA系统进行体能状态评估。评分为Fit（健康）且符合auto-HSCT条件的患者可进行auto-HSCT，不符合auto-HSCT条件的患者按照上述推荐的原则选择方案；评分为Intermediate-fitness（中等健康）的患者可选择强度减低的三药联合化疗；评分为Frail（虚弱）的患者，建议选择以CD38单克隆抗体为基础的三药联合化疗。CAR-T细胞和BsAb在>70岁及虚弱患者中仍可安全应用。

（3）高危年轻MM患者：如有合适供者，可在疾病再诱导缓解后考虑异基因造血干细胞移植。

3. 根据患者既往用药/耐药情况推荐治疗方案：

（1）来那度胺耐药治疗方案（证据来源）：

·DVd/IsaVd方案：CD38单克隆抗体+硼替佐米+地塞米松（CASTOR/LEPUS Ⅲ期[23]–[24]）；

·DKd/IsaKd方案：CD38单克隆抗体+卡非佐米+地塞米松（CANDOR Ⅲ期，IKEMA Ⅲ期[25]–[26]）；

·DPd/IsaPd方案：CD38单克隆抗体+泊马度胺+地塞米松（APOLLO Ⅲ期，ICARIA Ⅲ期[27]–[28]）；

·PVd方案：泊马度胺+硼替佐米+地塞米松（OPTIMISMM Ⅲ期[29]）；

·SVd方案：塞利尼索+硼替佐米+地塞米松（BOSTON Ⅲ期[30]）；

·KPd方案：卡非佐米+泊马度胺+地塞米松（EMN011/HO114/SELECT，Ⅰ/Ⅱ期[31]–[32]）；

·SPd方案：塞利尼索+泊马度胺+地塞米松（STOMP Ⅰb/Ⅱ期[33]）；

·SKd方案：塞利尼索+卡非佐米+地塞米松（STOMP Ⅰb/Ⅱ期[33]）；

·SDd方案：塞利尼索+CD38单克隆抗体+地塞米松（STOMP Ⅰb/Ⅱ期[33]）。

（2）硼替佐米耐药治疗方案（证据来源）：

·DRd/IsaRd方案：CD38单克隆抗体+来那度胺+地塞米松（POLLUX Ⅲ期[34]）；

·DKd/IsaKd方案：CD38单克隆抗体+卡非佐米+地塞米松（CANDOR Ⅲ期，IKEMA Ⅲ期[25]–[26]）；

·DPd/IsaPd方案：CD38单克隆抗体+泊马度胺+地塞米松（APOLLO Ⅲ期，ICARIA Ⅲ期[27]–[28]）；

·KRd方案：卡非佐米+来那度胺+地塞米松（ASPIRE Ⅲ期[35]）；

·KPd方案：卡非佐米+泊马度胺+地塞米松（EMN011/HO114/SELECT，Ⅰ/Ⅱ期[31]–[32]）；

·SKd方案：塞利尼索+卡非佐米+地塞米松（STOMP Ⅰb/Ⅱ期[33]）；

·SPd方案：塞利尼索+泊马度胺+地塞米松（STOMP Ⅰb/Ⅱ期[33]）；

·SDd方案：塞利尼索+CD38单克隆抗体+地塞米松（STOMP Ⅰb/Ⅱ期[33]）。

（3）双重（来那度胺和硼替佐米）耐药治疗方案（证据来源）：

·DKd/IsaKd方案：CD38单克隆抗体+卡非佐米+地塞米松（CANDOR Ⅲ期，IKEMA Ⅲ期[25]–[26]）；

·DPd/IsaPd方案：CD38单克隆抗体+泊马度胺+地塞米松（APOLLO Ⅲ期，ICARIA Ⅲ期[27]–[28]）；

·KPd方案：卡非佐米+泊马度胺+地塞米松（EMN011/HO114/SELECT，Ⅰ/Ⅱ期[31]–[32]）；

·SKd方案：塞利尼索+卡非佐米+地塞米松（STOMP，Ⅰb/Ⅱ期[33]）；

·SPd方案：塞利尼索+泊马度胺+地塞米松（STOMP，Ⅰb/Ⅱ期[33]）；

·SDd方案：塞利尼索+CD38单克隆抗体+地塞米松（STOMP，Ⅰb/Ⅱ期[33]）。

（4）三重暴露/耐药（CD38单克隆抗体、硼替佐米、来那度胺耐药）治疗方案（证据来源）：

·KPd方案：卡非佐米+泊马度胺+地塞米松（EMN011/HO114/SELECT，Ⅰ期/Ⅱ期[31]–[32]）；

·SPd方案：塞利尼索+泊马度胺+地塞米松（STOMP Ⅰb/Ⅱ期[33]）；

·SKd方案：塞利尼索+卡非佐米+地塞米松（STOMP Ⅰb/Ⅱ期[33]）；

·CAR-T细胞治疗：伊基奥仑赛、泽沃基奥仑赛（FUMANBA-1 Ⅱ期、LUMMICAR Ⅰ/Ⅱ期、KarMMa-3 Ⅲ期、CARTITUDE-4 Ⅲ期[36]–[39]）；

·BsAb：特立妥双抗、埃纳妥双抗、塔奎妥双抗（MajesTEC-1，MagnetisMM-3，MonumenTAL-1，Ⅰ/Ⅱ期[40]–[42]）。
